# Prevalence, Distribution, and Factors Associated with Vector-Borne Pathogen Infections in Pet Dogs from Different Geoclimatic Zones in Sri Lanka

**DOI:** 10.1155/2023/9467314

**Published:** 2023-11-13

**Authors:** Ushani Atapattu, Vito Colella, Andrew Worsley, Lucas G. Huggins, Anke Wiethoelter, Rebecca J. Traub

**Affiliations:** Faculty of Science, University of Melbourne, Melbourne, VIC 3010, Australia

## Abstract

Vector-borne pathogens (VBPs) cause significant diseases in dogs in the tropics. In Sri Lanka, the scarce availability of previous studies on canine VBPs has hampered an accurate evaluation of their prevalence in pet dog populations. In this study, we collected demographic, clinical, and environmental data together with whole blood from 423 pet dogs from three geoclimatic zones in Sri Lanka. All blood samples were screened using a previously validated multiplex qPCR assay to detect the six most prevalent canine VBPs in tropical Asia. Multivariable logistic regression models were used to investigate environmental and host factors as predictors of VBP infections. Overall, 254 dogs (60.1%, 95% CI: 55.3–64.6%) were infected with one or more VBPs. *Babesia gibsoni* was the most prevalent VBP with 37.4% (95% CI: 32.7–42.2%) of dogs infected followed by *Hepatozoon canis* (21.04%, 95% CI: 17.25–25.24%), haemotropic mycoplasmas (10.2%, 95% CI: 7.5–13.4%), *Babesia vogeli* (5%, 95% CI: 3.2–7.5%), *Ehrlichia canis* (4.5%, 95% CI: 2.7–6.9%), and *Anaplasma platys* (3.8%, 95% CI: 2.12–6.1%). Predictors of VBP infections included tick infestation for *H. canis* (*p*=0.05) and *A. platys* (*p*=0.01), as well as age for *B. gibsoni* (*p*=0.01) and *H. canis* (*p*=0.05) infection. Local breed (*p*=0.004), male dogs (*p*=0.001) and flea infestation (*p*=0.04) were significantly associated with haemotropic mycoplasma infections suggesting direct-blood exchange through fighting and fleas as a possible means of transmission for these pathogens. Clinical results suggest that *B. gibsoni* and *E. canis* caused clinically significant disease, especially in exotic breeds such as German shepherds and Rottweilers compared to the local breeds (p < 0.001). Measures such as educating pet dog owners on the importance of being vigilant on ectoparasite infestation of their pets, preventing pet dogs from interacting with stray or community dogs, and the compliant use of effective ectoparasiticides will be crucial for effective control of VBPs in pet dogs in Sri Lanka.

## 1. Introduction

Vector-borne pathogens (VBPs), *Babesia gibsoni*, *Babesia vogeli*, *Hepatozoon canis*, *Ehrlichia canis*, *Anaplasma platys*, and potentially haemotropic mycoplasmas are canine blood-borne pathogens transmitted by tick-vectors that have been identified as ubiquitous across tropical Asia [1–4]. Infection with these pathogens can result in significant clinical disease from the direct effects of the pathogen itself and the host immune response with potentially fatal consequences. *Babesia gibsoni* and *E. canis* can cause severe pathology, whereas subclinical infection predominates in *B. vogeli*, *H. canis*, and haemotropic mycoplasma infections. However, all these VBPs can cause significant disease in immunocompromised individuals and can result in complex pathologies when present as coinfections [5].

Infections with these VBPs are anecdotally known to be common throughout Sri Lanka. However, to date, a limited number of studies in dogs have demonstrated *B. gibsoni*, *B. canis* [6–8], *H. canis* [9, 10], *E. canis* [11, 12], and *Anaplasma* spp. [13] to be present. Nonetheless, a notable proportion of these publications were based on case reports [7–10]. Additionally, many of these studies [7, 8, 10, 14] utilised microscopic-based methods for diagnosing VBP, which are notorious for their low sensitivity [15]. Only one study utilised molecular assays to detect the prevalence of *E. canis* in stray dogs residing within the Colombo district [11], and another utilised immunodiagnostic assays for the seroprevalence of *Anaplasma* spp. and *E. canis* in Kalutara district [13], Sri Lanka. To date, predictors for VBP infections and their clinical impact on dogs residing in Sri Lanka have not been assessed limiting the translational value of the previous studies.

Here, we conduct a comprehensive, island-wide, cross-sectional study using high-throughput molecular diagnostics to assess the prevalence and predictors associated with VBP infection in Sri Lankan pet dogs. Such data will assist in identifying the clinical significance of VBP infection and facilitate better diagnosis, treatment, and control of these pathogens.

## 2. Materials and Methods

### 2.1. Study Sites and Sampling

**Figure 1:**
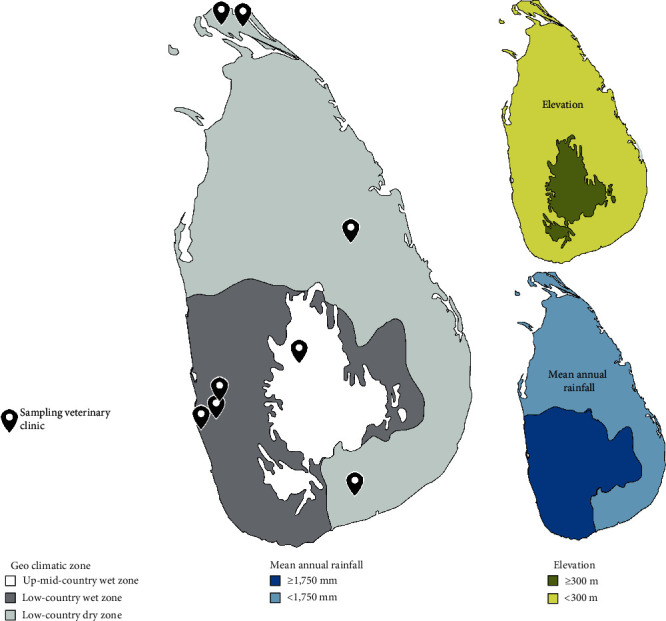
A schematic diagram of Sri Lanka indicating sampling locations and the geoclimatic zones, mean annual rainfall, and elevation across the island (modified from Department of Meteorology, Sri Lanka, 2019 and Free Vector Maps, 2022 (https://freevectormaps.com/sri-lanka/LK-EPS-01-0002?ref=atr) using Adobe illustrator version 26.5, 2022).

Sri Lanka, located between 5°55′ and 9°51′N latitude and 79°41′ and 81°53′E longitude, is an island nation with a tropical climate. The country can be divided into three climatic zones based on mean annual rainfall and geographical relief: the low-country dry zone, the low-country wet zone, and the mid-up country wet zone (Figure 1). The wet zone receives an average annual rainfall of over 1,750 mm, while the dry zone receives less than 1,750 mm [16]. Additionally, areas above 300m elevation are classified as the mid-up country, and those below 300 m are considered the low country [16]. Dogs in Sri Lanka can be largely categorised into two groups as unowned (stray) or owned (pets). Stray dogs comprise the local dog breed and are exclusively free roaming. Owned dogs on the other hand are of local or exotic breeds such as Rottweilers and German Shepherds and their crosses and may be restricted within the confines of their owners' properties or allowed to roam unsupervised. Close contact and physical interactions between these stray and pet categories of dogs are common across the island. The average population density of 384 people per km^2^ [17] in Sri Lanka was recorded to be proportional to the dog population density in several studies [18–20], meaning that a higher number of dogs resides in densely populated regions of the country.

Accordingly, eight veterinary clinics/hospitals across the three geoclimatic zones (Figure 1) were selected based on veterinarians' willingness to participate in the study. Within each clinic, animals were randomly recruited, with exception of dogs requiring emergency attention. Where applicable, only one dog per owner was selected for inclusion in the study.

Sample size calculations were based on either estimating the prevalence of each VBP with 95% confidence or demonstrating freedom from the pathogen, whichever required the larger sample number, assuming a test sensitivity of 75% and specificity of 95% using the Shiny applet developed using the “epiR” package [21] and “Epitools” web platform (https://epitools.ausvet.com.au/), respectively (*Supplementary 1*).

### 2.2. Collection of Data

Data were collected during a 11-month period from April 2020 to March 2021. Following owner consent, individual animal metadata including age, sex, neutering status, breed, history of vector-borne diseases as diagnosed by their veterinarian, as well as frequency of ectoparasitic treatment, “brand” (formulation), frequency of use, and duration from the last ectoparasiticide treatment given were collected from the owner of each participating dog to the best of their knowledge using a structured questionnaire (*Supplementary 2*).

Clinical manifestations such as poor body condition, presence of palpable splenomegaly and hepatomegaly, mucous membrane colour, body temperature, and palpable lymph node enlargement, were obtained through a physical examination by a veterinarian. Examination of the whole-body surface (including interdigital spaces) for ∼5 min was performed to identify tick, flea, and/or louse infestation. If at least one tick, flea, and/or louse was found, the pet was considered to have an active tick, flea, and/or louse infestation. The dog's body condition was assessed according to the WSAVA five scale BCS (body condition score) chart [22] and those with BCS <3 was categorised as having “low” BCS.

From each dog, individual whole blood samples (1–2 ml) were collected in a single sterile EDTA (ethylenediaminetetraacetic acid) tube through cephalic or lateral saphenous venepuncture. The collected blood samples were transported on ice and were stored at −20°C until DNA was extracted.

### 2.3. DNA Extraction and Molecular Screening

Extraction of DNA from canine whole-blood was performed using the DNeasy Blood and Tissue Kit (Qiagen, Hilden, Germany) according to the manufacturer's protocol at the University of Peradeniya, Sri Lanka. The extracted DNA was stored at −20°C until shipped to the University of Melbourne, Australia. The extracted DNA was screened in duplicates using a previously developed TaqMan® probe-based multiplex real-time PCR assay comprising two separate fourplex reactions for vector-borne bacteria and protozoa, respectively [23]. Together these fourplex reactions are able to detect six common VBPs targeting a partial region of the 18S ribosomal RNA gene for the protozoans *B. gibsoni*, *B. vogeli*, *H. canis*, and a hypervariable region of the 16S rRNA gene for the bacteria *E. canis*, *A. platys*, and haemotropic mycoplasmas, viz. *Mycoplasma haemocanis*, *Candidatu*s Mycoplasma haemominutum, and *Candidatus Mycoplasma haematoparvum*. The optimised PCR assays were run as 10 *µ*l reactions using QuantiNova® Probe PCR Master Mix (Qiagen, Hilden, Germany) with 1 *µ*l of template DNA in 96-well skirted white plates (Qiagen, Hilden, Germany) using a QIAquant 96 5plex real-time PCR thermal cycler (Qiagen, Hilden, Germany). Mammalian mitochondrial DNA and equine herpes virus DNA were used as extraction and internal controls, respectively, along with a no template reaction control (NTC). Appropriate gBlocks™ synthetic double-stranded DNA sequences (Integrated DNA Technologies, USA) of the pathogens tested were used as positive controls. The samples that had achieved endpoint (plateau phase) on or before the last two cycles and had a Ct lower or equal to 35 cycles in both replicates were considered positive.

### 2.4. Data Analysis

Data were checked and cleaned for statistical analysis with Microsoft Excel® for Microsoft 365 MSO (Version 2212 Build 16.0.15928.20196) and R version 4.2.0 [24] using “dplyr” [25] and “janitor” [26] packages. For descriptive and univariable analysis, dogs were categorised in to six age groups according to Harvey et al. [27] and two breed categories: local and exotics. Dogs exhibiting one or more signs from pale mucous membranes, reduced appetite/anorexia, fever, lymphadenomegaly, splenomegaly and hepatomegaly were considered as having clinical signs of uncomplicated cases of VBP infections [28]. The compliance to ectoparasiticides by owners of each study dog were based on whether they were being treated for ectoparasites, whether the frequency and duration of treatment were compatible with the product used, and whether the approximate duration since the last treatment adhered to the recommended frequency for the product used. The scoring protocol for each scenario is indicated in *Supplementary 1*. With this scoring, a score of 3 corresponds to a compliant owner, and those with minimal and moderate compliance received a score of 1 and 2, respectively. Owners with poor compliance with ectoparasite treatment and untreated dogs received a score of zero.

Univariable associations of VBP infection and host factors, i.e., age, breed, sex, neuter status, presence of ticks/fleas, and other related factors, i.e., geoclimatic zone, and clinical manifestations were assessed using binomial logistic regression with generalised linear model (GLM), Pearson *χ*^2^ test or Fischer's exact test, based on the structure of data using R version 4.2.0 [24]. The odds ratios were calculated with 95% confidence using “oddsratio” function of the “epitools” package [29]. Variables used in fitting multivariable models (*Supplementary 1*) were compared in pairs using the Cremers V test using “rcompanion” package [30] in R to determine the degree of correlation before including them in models to omit collinearity.

Random effects logistic and ordinal regression analyses were employed to analyse the risk of infection with individual pathogen and with one or more infections, respectively. All selected variables (*Supplementary 1*) were included as fixed effects in the generalised linear mixed-effect models (GLMM) and sampling veterinary clinics were used as random effects using “lme4” package [31] in R. Ordered proportional odds logistic regression (with ordered logit model) was performed with “MASS” [32] package in R. The “sjPlot” [33] package in R was used to calculate odds ratios for the models and to visualise models where necessary. Selection of the models were performed by the backward stepwise elimination to find the best fit [34]. The Brent–Wald test in “brant” package [35] in R was used to determine whether the proportional odds assumption holds for the ordered proportional odds logistic regression model [36], where the Brent–Wald test output is *p* ≥ 0.05 for the final selected model variables, the model was considered to hold the proportional odds assumption. The R code for the functions used in this study is available at https://github.com/ushata/Tick-borne-pathogens-SL-dogs.

## 3. Results

### 3.1. Sample Descriptives

A total of 423 blood samples were collected from pet dogs from the up-mid country wet zone (*n* = 307), low-country wet zone (*n* = 75), and low-country dry zone (*n* = 41) in Sri Lanka. More than half of the dogs were local breed dogs and their crosses (*n* = 265), while the rest were of exotic breeds (*n* = 147). Of the dogs presented, 45.2% were healthy and were presented to the veterinarian for routine vaccination, health checks, or neutering. Data on age group, sex, neutering status, breed, tick, flea, and louse infestation, and ectoparasiticide usage of the study cohort according to the geoclimatic zone are summarised in *Supplementary 1*.

### 3.2. Prevalence and Univariable Associations on Vector-Borne Pathogen Infections and Host and Environmental Variables

**Figure 2:**
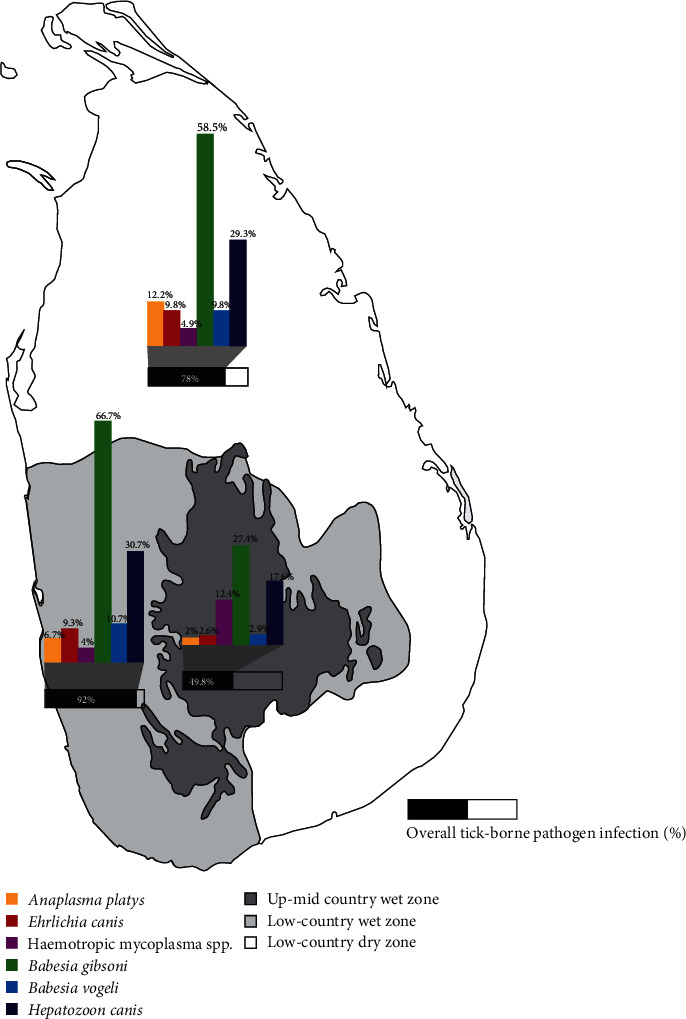
Prevalence and distribution of tick-borne pathogen infections across the three geoclimatic zones in Sri Lanka.

All six VBPs investigated were found in Sri Lankan dogs. Overall, 60% (95% CI: 55–65%) of dogs were infected with at least one VBP. *Babesia gibsoni* was found in 37% (95% CI: 33–42%) of dogs, being the most prevalent pathogen among the study subjects, followed by *H. canis* (21%, 95% CI: 17–25%), haemotropic mycoplasmas (10%, 95% CI: 8–13%), *B. vogeli* (5%, 95% CI: 3–8%), *E. canis* (5%, 95% CI: 3–7%), and *A. platys* (4%, 95% CI: 2–6%) (Figure 2). Mixed VBP infections were found in 31% (95% CI: 26–37%) of those infected. Results of single and mixed VBPs infections are summarised in Table 1. The VBP infection results according to age group, sex, neutering status, breed group, tick, flea, and louse infestation, and ectoparasiticide treatment status and their univariable associations are summarised in Tables 2 and 3, respectively. Dogs living in the low country were more likely to be infected with VBPs than those in the mid and up-country wet zone (p < 0.001). Conversely, infection by haemotropic mycoplasmas was not significantly influenced by geoclimatic zones [37] but tended to occur more commonly in dogs in the mid-up-country wet zone (Table 3).

### 3.3. Use of Ectoparasiticides

Nearly half (*n* = 189, 44.7%) of the participating dog owners claimed to use ectoparasiticides or repellents to control ticks, fleas, and lice, while nearly 35% (*n* = 145) did not (Table 4). The remaining dog owners were unaware whether their pets had been administered treatment for ectoparasite control. Propoxur powder (30.8%, *n* = 60), afoxolaner tablets (18.97%, *n* = 37), and subcutaneous ivermectin injections (10.3%, *n* = 20) were the most common ectoparasiticides or repellents used by the study subjects (Table 4). Of those who claimed to treat their pets with ectoparasiticides, 55.3% (*n* = 169) were minimally compliant with the recommended treatment protocol for effective ectoparasite control for their preferred product, and 4.5% (*n* = 19) showed moderate degree of compliance. Only one of 189 dogs that received ectoparasiticides demonstrated recommended treatment compliance (Table 4).

### 3.4. Association of Vector-Borne Pathogen Infections with Clinical Manifestations

The univariable statistical associations of VBP infections and clinical manifestations are summarised in Table 5. Of the 423 examined dogs, 34% (95% CI: 30–39%) exhibited one or more clinical signs associated with uncomplicated cases of VBP infections [28]. From these 67% (95% CI: 59–74%) were identified as infected with one or more VBPs and constituted 38% (95% CI: 32–44%) of the total number of infected animals. Exotic breeds were more likely to exhibit clinical signs associated with VBP disease than local dogs (OR 2.52, 95% CI: 1.32–4.78, *p*=0.02). Mixed infections with two or more VBPs tended to be more clinically noticeable (OR 1.96, 95% CI: 1.13–3.41, *p*=0.05) compared to mono-infections (Table 4). Pale mucosae (OR 1.58, 95% CI: 0.97–2.57, *p*=0.063), palpable peripheral lymphadenomegaly (OR 2.50, 95% CI: 0.98–6.39, *p*=0.048), poor body condition (OR 1.60, 95% CI: 0.92–2.78, *p*=0.093), and reduced appetite/anorexia (OR 2.70, 95% CI: 0.91–8, *p*=0.07) were some of the clinical signs associated with the presence of at least one VBP. Pale mucosae were significantly associated with dogs infected with *B. gibsoni* (OR 1.64, 95% CI: 1.03–2.62, *p*=0.036) and peripheral lymphadenomegaly was significantly associated with *E. canis* infection (OR 4.1, 95% CI: 1.3–13.4, *p*=0.034). Dogs with wounds (OR 2.7, 95% CI: 1.2–6, *p*=0.04) were more likely to be infected with haemotropic mycoplasmas (Table 5).

Pet dogs with a previous history of vector-borne disease 11% (95% CI: 7.7–15%) were more likely infected with a VBP than those without a history of vector-borne disease (*n* = 31, OR 6.83, 95% CI: 2.36–19.86, p < 0.001). The majority of these dogs 65% (95% CI: 45–81%) were infected with *B. gibsoni* either alone or coinfected with the other VBPs.

### 3.5. Multivariable Models Predicting Risk Factors for Vector-Borne Infections in Dogs

The coefficient estimates, odds ratios, and *p*-values for our GLMMs and ordered logistic regression model are summarised in Table 6. On average, tick infestation significantly increased the odds of an infection with *A. platys* (OR 4.8, 95% CI: 1.38–16.65, *p*=0.013) and *H. canis* (OR 1.73, 95% CI: 1.01–2.96, *p*=0.047) when other variables were held constant. Odds for overall VBP infection (OR 1.1, 95% CI 1.04–1.17, *p*=0.002) for infection with *B. gibsoni* (OR 1.08, 95% CI: 1.02–1.14, *p*=0.01) and *H. canis* (OR 1.07, 95% CI: 1.01–1.14, *p*=0.034) increased with increasing age on average when other variables were held constant. For haemotropic mycoplasmas, male sex (OR 4.21, 95% CI: 1.85–9.57, *p*=0.001), local breed (OR 4.33, 95% CI: 1.62–11.57, *p*=0.004), and active flea infestation (OR 2.34, 95% CI: 1.05–19, *p*=0.037) were predictors of infection on average holding all other variables constant (Table 5). For *A. platy*s, an active louse infestation (OR 23.36, 95% CI: 3.34–163.44, *p*=0.002) was another predictor on average holding all other variables constant. No significant predictors were identified for *E. canis* and *B. vogeli*. *Hepatozoon canis* infection was more prevalent in the low-country wet zone (OR 2.41, 95% CI: 1.32–4.43, *p*=0.004 and OR 3.95, 95% CI: 1.14–13.66, *p*=0.03, respectively) compared to the other two geoclimatic zones. Ordered logistic regression model on possible predictors for mono- or mixed infections identified the odds of mixed infections to increase with age (OR 1.08, 95% CI: 1.03–1.14, *p*=0.003), active flea infestation (OR 1.7, 95% CI: 1.12–2.57, *p*=0.012) and if the dog resided in the low-country wet (OR 5.79, 95% CI: 3.36–10.18, p < 0.001) and low-country dry (OR 6.8, 95% CI: 3.17–14.96, p < 0.001) zones on average holding all other variables constant (Table 6).

## 4. Discussion

This study reports the first comprehensive survey on the prevalence and predictors for canine VBPs in owned dogs in Sri Lanka. The study highlights the highly endemic nature of these VBPs, especially with respect to *B. gibsoni* and provides the first molecular evidence for the presence of canine haemotropic mycoplasmas and *A. platys* in Sri Lankan dogs. In addition, this study attributes *B. vogeli* as the large *Babesia* species infecting dogs in Sri Lanka, which has to date, been likely misclassified as *B. canis* using light microscopy [7, 14]. This study revealed that over a third of owned dogs in Sri Lanka were positive for *B. gibsoni* compared to 15% detected by Weerathunga et al. [14] using light microscopy in Anuradhapura district. The prevalence of *E. canis* in owned dogs in Sri Lanka at 4.5% was significantly lower than the previous 17% reported for ‘apparently healthy' stray dogs in the country using nested PCR [11] and 24.5% using microscopy by Weerathunga et al. [14]. This discrepancy might be explained due to differences in study cohorts (owned versus stray dogs) in the former and the low specificity (high-false positivity rate) of microscopic identification of *E. canis* morulae in the latter. Identification of *Ehrlichia* morulae through light microscopy is challenging as they are transient in the blood and low in number [38] and can be confused with cells granules [39], contributing towards false positive results. With the exception of *H. canis*, the prevalence of the *Rhipicephalus linneai*-transmitted pathogens, *B. vogeli*, *E. canis*, and *A. platys* in this study were significantly lower than those reported in the surveys of stray dogs in neighbouring India [1, 2]. The prevalence of *H. canis* and haemotropic mycoplasmas in owned dogs in Sri Lanka are comparable to those reported in stray dogs in India [1].

Owing to COVID-19 related interprovincial travel restrictions, samples were sourced from only eight veterinary clinics/hospitals, with the majority sourced from a single geoclimatic zone, which may have introduced sampling and selection bias. However, we collected beyond the minimum number of samples required for the study and detected all six anticipated VBPs, even in pet dogs that received veterinary care. Therefore, despite the limitations, this study provides robust evidence of the burden of VBP in dogs in Sri Lanka and potential predictors of infection. Historical information, such as ectoparasiticide usage and previous VBP pathogen infection, may be influenced by recall and behavioural biases from pet owners and inclusion of dogs seen in veterinary clinics/hospitals in this prevented the assessment of VBP infection rates in dogs not regularly receiving veterinary care.

The multivariable analysis indicated local breeds and male dogs with observable flea infestation as a predictor for haemotropic mycoplasma infection compared with female dogs and exotic breeds without flea infestation. A higher prevalence of haemotropic mycoplasmas has been demonstrated to associated with fighting dogs [40–42] and those housed in kennels [42–44], where dog-to-dog contact is frequent. Although not significant, there was a positive correlation between dogs presenting with wounds, predominantly caused by dog bites, and haemotropic mycoplasma infection, further indicating dog fighting as a likely mode of transmission for this group of pathogens. This may indicate that males, and local dog breed that are less likely to be confined at home by their owner, may be at greater risk of engaging in dog fights and acquiring haemotropic mycoplasma infection. In addition to fighting, higher blood concentrations of androgenic hormones in entire male can also contribute to poorer immunity, which may increase their susceptibility to haemotropic mycoplasma infection [45, 46].

The transmission of haemotropic mycoplasmas was initially proposed to be by *R. linnaei* [47], but without convincing evidence. Fleas, on the other hand, are known vectors for *Mycoplasma haemofelis* [48], a closely related feline-specific species to *Mycoplasma haemocanis* [49, 50]. Recently, transmission of haemotropic mycoplasma has been demonstrated in a population of dogs on ectoparasiticides and in the absence of arthropod vectors, strongly suggesting non-vectorial transmission for these pathogens [42]. Interestingly, the canine haemotropic mycoplasmas were more likely to occur in dogs residing in the up-mid-country wet zone in contrast to all five vector-borne pathogens that were significantly more common in dogs in the low-country wet and dry zones. The up-mid-country wet zone experiences mean annual temperatures between 10°C and 25°C [51] and relative humidity between 55% and 90%, compared to higher mean annual temperatures (25°C–30°C) experienced in the low country wet and dry zones. According to Silverman et al. [52], the life span of 90% of unfed *Ctenocephalides felis* adults on average is between 8–22 days in temperature and humidity ranges of up-mid-country wet zone as opposed to 2–8 days in that corresponding to low country wet and dry zones [52]. Furthermore, 61% (95% CI: 55–66%) of the dogs in the up-mid country in our study were infested with fleas compared to 40% (95% CI: 30–51%) in the low country. Nevertheless, in a previous study conducted in south-east Asia, neither flea (mainly *Ctenocephalides felis*) infestation was correlated with haemotropic mycoplasmas infection nor were these pathogens found in fleas collected from infected dogs [53].

We identified that the risk of infection of *B. gibsoni* increases with age. Possible explanations for such an outcome are multifactorial. The multimodal nature of transmission of *B. gibsoni* through its tick vector, direct infection through infected blood exchange (e.g., during dog fights [54] and blood transfusion), and transplacental transmission [55] can lead to frequent infection of dogs by this pathogen, with the former factors being cumulative with age. Furthermore, treatment of *B. gibsoni* usually fails in the complete elimination of the pathogen [56], even when the recommended azithromycin–atovaquone combination [57] is used, causing most dogs to remain subclinically infected for life. Given the study population's characteristics in Sri Lanka, dog fighting is also likely to play an essential role in transmitting this pathogen. However, transplacental transmission appears to be absent or negligible, given that in this study, most pups of age less than 6 months remained uninfected despite the high prevalence of these pathogens in adult dogs.

Conversely, for *A. platys* and *H. canis*, known to be transmitted by *R. linnaei* bites and ingestion, respectively, tick infestation was identified as a significant predictor of infection. An active tick infestation can increase the likelihood of tick ingestion and in turn, *H. canis* infection. In addition, the risk of *H. canis* infection increased with animal age. Additionally, louse infestation appears to be a predictor for *A. platys* infection. Owing to low prevalence of louse infestations these results are of limited value, although *A. platys* DNA was previously detected in lice from pups in Australia [58].

Treatment of *H. canis* is likely missed in most instances as the majority of infections are subclinical and, therefore, owners are less likely to seek veterinary treatment. Even if infected dogs are presented for treatment, it is necessary to administer an intensive treatment regime of fortnightly imidocarb dipropionate until elimination of the pathogen is achieved, which most pet owners are less likely to comply with, causing a persistent infection, which is likely to accumulate on a population level over time [59]. For *E. canis* and *B. vogeli*, no significant predictors of infection were identified in our models. Even though the real-time PCR assay is reported to have over 95% diagnostic sensitivity [23], noncirculating, subclinical infections of VBPs, as well as latent or chronic (pancytopenic) phases of *E. canis* infections, could lead to under-reporting of the true prevalence of these pathogens [60].

Most veterinary clinics in Sri Lanka are not equipped with resources to perform haematological, biochemical, or PCR-based tests to diagnose VBP infections [61], with veterinarians relying on clinical presentation rather than proactively investigating the presence of VBPs in dogs exhibiting subclinical signs. This is noteworthy as more than half of the VBP infections in our study were subclinical or clinically unremarkable. Subclinical infections can act as a “ticking time bomb,” for example in cases of latent ehrlichiosis [62] or in instances where VBPs can reactivate to cause acute disease during times of natural or iatrogenic immunosuppression [55, 63, 64]. In addition, subclinically infected dogs can act as a continual source of infection for other dogs. Therefore, where possible, best practice recommendations should be followed by Sri Lankan veterinarians to screen and treat for VBPs, in particular *B. gibsoni*, in dogs presenting with a history of roaming, fighting and/or tick exposure, regardless of whether overt clinical signs are present.

Even though no breed-based differences were identified for VBP infection, exotic breeds were more likely to display clinical signs of VBP infection on physical examination compared to local dogs. There is no concrete evidence to explain this disparity, but available data suggest that host immunological and genetic factors may be responsible for this difference [65, 66]. In this study, pale mucosae indicating anaemia was the most significant clinical manifestations of *B. gibsoni* infection, while reduced or loss of appetite and presence of peripheral lymphadenomegaly were significant to a lesser extent. For *E. canis*, peripheral lymphadenopathy was associated with infection whereas pale mucosae and poor body condition were associated to a lesser extent. Peripheral lymphadenomegaly resulting from increased proliferative activities in the lymph nodes by *E. canis* antigens [67] is a well-known clinical sign during acute canine ehrlichiosis. Severe bone marrow suppression and haemolysis are potentially fatal manifestations in dogs with *E. canis* and *B. gibsoni*, which are likely complicated by host immune responses [68, 69]. Infections with *B. vogeli*, *H. canis*, *A. platys*, and haemotropic mycoplasmas did not demonstrate clinically overt disease. Noninfectious causes (e.g., heart-, hepatic- or kidney-disease, neoplasia, etc.) exacerbate clinicopathological manifestations of VBPs, but so can the presence of coinfections with other VBPs [5]. Indeed, there was an association between clinical signs and multiple VBP infections (*p*=0.054).

Year-round tick control using an ectoparasiticide product that both repels and kills ectoparasites prior to feeding is the mainstay of preventing VBP infections in dogs in the tropics [70, 71]. The vast majority of owners used topical short-acting preparations that required a high frequency of administration to be effective (e.g., propoxur-based powders, pyrethroid-, amitraz-based shampoos), or that lacked proven ectoparasiticidal efficacy (e.g., herbal shampoos) [72]. In addition, propoxur-resistant ticks have been identified in Sri Lankan cattle [73], but its efficacy against local *R. linnaei* populations remains unknown. Almost a fifth of dog owners used systemically acting products that require ectoparasite feeding to occur prior to kill (e.g., isoxazolines). Only a minority of pet owners used fipronil-based products that have proven efficacy for the prevention of VBPs in the tropics [71]. Regardless of ectoparasiticide product of choice, almost all the owners did not follow the recommended treatment schedule for ectoparasiticides. In contrast, over 60% of the dog owners of high-income nations such as the United States of America are aware of the veterinary recommendations for the prevention of ticks and fleas on their pets [74].

These factors have likely contributed to the high prevalence of VBP infection observed in pet dogs in Sri Lanka, even in animals receiving ectoparasiticide treatment. Client choice of ectoparasiticide treatment is not only dependent on affordability, but also on product availabiity given that many effective commercial ectoparasiticides are not registered in Sri Lanka [61]. These results highlight the need for veterinarians to follow best practice guidelines when advising clients on both ectoparasiticide choice and the importance of strictly following labelled ectoparasiticide treatment frequency for the prevention of VBP in Sri Lanka.

## 5. Conclusions

This study comprehensively reports the prevalence and associated factors for VBP infections in Sri Lankan pet dogs and identifies key gaps in their control. To prevent VBP infections, veterinarians should recommend appropriate and effective ectoparasiticides according to their labelled recommendations. Sri Lankan veterinarians should \provide dog owners with information on practices that would minimise the transmission risk for *B. gibsoni* and haemotropic mycoplasmas and be cautious when using canine blood for transfusions. Screening for VBP infections is recommended in such instances, preferably using molecular diagnostics.

## Figures and Tables

**Table 1:** Prevalence of single and mixed vector-borne pathogen infections detected in dogs in this study.

Pathogen/s	*n*	%
Single infections		
* B. gibsoni*	102	40.2
* H. canis*	34	13.4
Haemotropic mycoplamas	19	7.5
* B. vogeli*	9	3.5
* E. canis*	7	2.8
* A. platys*	3	1.2
Mixed infections		
* B. gibsoni* + *H. canis*	28	11
Haemotropic mycoplamas + *B. gibsoni*	10	3.9
Haemotropic mycoplamas + *H. canis*	7	2.8
* E. canis + B. gibsoni*	6	2.4
* B. vogeli + H. canis*	6	2.4
* A. platys + B. gibsoni*	5	2
* A. platys + H. canis*	3	1.2
* A. platys + B. vogeli*	2	0.8
* E. canis + H. canis*	1	0.4
* E. canis + B. vogeli*	1	0.4
Haemotropic mycoplamas + *B. vogeli*	1	0.4
* E. canis + B. gibsoni + H. canis*	2	0.8
Haemotropic mycoplamas + *B. gibsoni + H. canis*	2	0.8
* A. platys + B. gibsoni + H. canis*	1	0.4
* A. platys* + Haemotropic mycoplamas + *B. gibsoni*	1	0.4
* A. platys + B. vogeli + H. canis*	1	0.4
Haemotropic mycoplamas + *E. canis* + *H. canis*	2	0.8
Haemotropic mycoplamas + *B. gibsoni* + *B. vogeli + H. canis*	1	0.4
Total infections	254	

**Table 2:** Single and mixed vector-borne pathogen infection in dogs in Sri Lanka according to geoclimatic zone, age group, sex, neutering status, breed group, tick, flea, louse infestation, and ectoparasiticide treatment.

		Pathogen positives (%)							Infection type (%)	
	Total	Overall	*A. platys*	*E. canis*	Haemotropic mycoplasmas	*B. gibsoni*	*B. vogeli*	*H. canis*	Single infection	Mixed infection
Geoclimatic zone										
Up-mid-country wet zone	307	49.8	2	2.6	12.4	27.4	2.9	17.6	36.5	13.4
Low-country wet zone	75	92	6.7	9.3	4	66.7	10.7	30.7	61.3	30.7
Low-country dry zone	41	78.1	12.2	9.8	4.9	58.5	9.8	29.3	41.5	36.6
Breed										
Exotic	147	66	3.4	6.8	3.4	42.2	6.1	18.4	53.1	12.9
Local	264	58	4.2	3.4	14.4	35	4.5	23.0	35.6	22.3
Unknown	13	30.8	0	0	0	33.3	0	9.1	25	8.3
Neuter status										
Intact	312	60.3	3.8	5.8	11.9	37.2	5.4	20.1	39.7	20.5
Neutered	81	61.7	3.7	1.2	7.4	35.8	2.5	27.2	48.1	13.6
Unknown	30	53.3	3.3	0	0	43.3	6.7	13.8	40	13.3
Sex										
Female	189	55.6	3.7	3.2	4.2	36	3.2	19.1	43.4	12.2
Male	216	65.7	4.2	6	16.2	39.4	6	23.6	40.3	25.5
Unknown	18	38.9	0	0	0	27.8	11.1	11.1	27.8	11.1
Age group										
<6 months	29	31	6.9	0	3.4	13.8	3.4	10.3	27.6	3.4
6–24 months	81	55.5	4.1	6.2	9.6	32.2	6.8	18.5	37	18.5
>2–6 years	79	69.3	5.3	3.5	9.6	46.5	5.3	24.6	47.4	21.9
>6 years	75	66.4	1.8	4.4	13.3	40.7	1.8	25.7	46	20.4
Unknown	22	45.5	0	4.8	9.5	38.1	9.5	9.5	33.3	14.3
Tick infestation										
Absent	273	56.8	1.5	4	9.2	35.5	3.7	18	43.2	13.6
Present	121	66.1	8.3	6.6	12.4	36.4	7.4	29.8	38.8	27.3
Unknown	29	65.5	6.9	0	10.3	58.6	6.9	13.8	34.5	31
Flea infestation										
Absent	175	58.3	1.7	3.4	5.1	35.4	5.1	21.1	45.7	12.6
Present	219	60.7	5	5.9	14.2	36.1	4.6	21.9	38.4	22.4
Unknown	29	65.5	6.9	0	10.3	58.6	6.9	13.8	34.5	31
Louse infestation										
Absent	392	59.4	2.8	4.6	10.6	36.2	4.3	21.5	41.8	17.6
Present	5	100	60	20	0	40	40	40	40	60
Unknown	26	61.5	7.7	0	7.7	53.8	7.7	11.1	34.6	26.9
Ectoparasiticides										
Given	189	56.6	3.7	5.8	8.5	30.2	5.8	18	44.4	12.2
Not given	145	55.9	4.8	5.5	13.1	33.8	5.5	22.8	31	24.8
Unknown	89	74.2	2.3	0	9	58.4	2.3	24.7	51.7	22.5

**Table 3:** Univariable associations of vector-borne pathogen (VBP) infection with geoclimatic zone, age group, sex, breed group, neutering status, tick, flea, and louse infestation.

Pathogen/Variable	Estimate ± Std. error	Odds ratio (95% CI)	*P*-value
*A. platys*			
Age			
(Intercept)	−2.87 ± 0.37		
Age (months)	−0.01 ± 0.01	0.99 (0.98–1.00)	0.297
Sex			
(Intercept)	−3.26 ± 0.39		
Female	Reference		
Male	0.12 ± 0.51	1.13 (0.41–3.22)	0.812
Breed group			
(Intercept)	−3.14 ± 0.31		
Local	Reference		
Exotic	−0.21 ± 0.55	0.81 (0.25–2.27)	0.701
Neutering status			
(Intercept)	−3.26 ± 0.59		
Neutered	Reference		
Entire	0.04 ± 0.66	1.04 (0.32–4.65)	0.952
Tick infestation			
(Intercept)	−4.21 ± 0.5		
Absent	Reference		
Present	1.8 ± 0.6	6.06 (1.98– 22.46)	**0.003**
Flea infestation			
(Intercept)	−4.05 ± 0.58		
Absent	Reference		
Present	1.11 ± 0.66	3.03 (0.93–13.56)	0.093
Louse infestation			
(Intercept)	−3.54 ± 0.31		
Absent	Reference		
Present	3.95 ± 0.96	51.95 (7.89–426.35)	**<0.001**
Geoclimatic zone			
(Intercept)	−3.92 ± 0.41		
Up-mid-country wet zone	Reference		
Low-country wet zone	1.28 ± 0.62	3.58 (1.01–12.23)	**0.04**
Low-country dry zone	1.94 ± 0.63	6.97 (1.92–24.28)	**0.002**
*E. canis*			
Age (months)			
(Intercept)	−3.01 ± 0.36		
Age (months)	0 ± 0.01	1.00 (0.99–1.01)	0.849
Sex			
(Intercept)	−3.42 ± 0.41		
Female	Reference		
Male	0.67 ± 0.5	1.95 (0.76–5.66)	0.184
Breed group			
(Intercept)	−3.34 ± 0.34		
Local	Reference		
Exotic	0.73 ± 0.47	2.07 (0.81–5.32)	0.123
Neutering status			
(Intercept)	−4.38 ± 1.01		
Neutered	Reference		
Entire	1.59 ± 1.04	4.90 (0.99–88.78)	0.125
Tick infestation			
(Intercept)	−3.17 ± 0.31		
Absent	Reference		
Present	0.52 ± 0.48	1.69 (0.64–4.28)	0.274
Flea infestation			
(Intercept)	−3.34 ± 0.42		
Absent	Reference		
Present	0.58 ± 0.5	1.78 (0.69–5.15)	0.254
Louse infestation			
(Intercept)	−3.03 ± 0.24		
Absent	Reference		
Present	1.65 ± 1.14	5.19 (0.26–37.42)	0.15
Geoclimatic zone			
(Intercept)	−3.62 ± 0.36		
Up-mid-country wet zone	Reference		
Low-country wet zone	1.35 ± 0.53	3.85 (1.31–11.08)	**0.012**
Low-country dry zone	1.4 ± 0.64	4.04 (1.04–13.5)	**0.028**
Haemotropic mycoplasma spp.			
Age (months)			
(Intercept)	−2.49 ± 0.26		
Age (months)	0.01 ± 0	1.01 (1–1.01)	0.096
Sex			
(Intercept)	−3.12 ± 0.36		
Female	Reference		
Male	1.48 ± 0.41	4.38 (2.07–10.38)	**<0.001**
Breed group			
(Intercept)	−1.78 ± 0.18		
Local	Reference		
Exotic	−1.56 ± 0.49	0.21 (0.07–0.5)	**0.001**
Neutering status			
(Intercept)	−2.53 ± 0.42		
Neutered	Reference		
Entire	0.52 ± 0.46	1.68 (0.73–4.57)	0.257
Tick infestation			
(Intercept)	−2.29 ± 0.21		
Absent	Reference		
Present	0.34 ± 0.35	1.4 (0.7–2.74)	0.328
Flea infestation			
(Intercept)	−2.91 ± 0.34		
Absent	Reference		
Present	1.11 ± 0.39	3.04 (1.46–6.96)	**0.005**
Louse infestation			
(Intercept)	−2.15 ± 0.17		
Absent	Reference	–	
Present	−14.42 ± 1073.11		0.989
Geoclimatic zone			
(Intercept)	−1.96 ± 0.17		
Up-mid-country wet zone	Reference		
Low-country wet zone	−1.22 ± 0.61	0.29 (0.07–0.85)	**0.047**
Low-country dry zone	−1.01 ± 0.75	0.36 (0.06–1.25)	0.174
*B. gibsoni*			
Age (months)			
(Intercept)	−0.67 ± 0.16		
Age (months)	0 ± 0	1 (1–1.01)	0.212
Sex			
(Intercept)	−0.58 ± 0.15		
Female	Reference		
Male	0.14 ± 0.21	1.15 (0.77–1.73)	0.485
Breed group			
(Intercept)	−0.63 ± 0.13		
Local	Reference		
Exotic	0.31 ± 0.21	1.36 (0.9–2.06)	0.142
Neutering status			
(Intercept)	−0.58 ± 0.23		
Neutered	Reference		
Entire	0.06 ± 0.26	1.06 (0.64–1.78)	0.819
Tick infestation			
(Intercept)	−0.6 ± 0.13		
Absent	Reference		
Present	0.04 ± 0.23	1.04 (0.66–1.61)	0.874
Flea infestation			
(Intercept)	−0.6 ± 0.16		
Absent	Reference		
Present	0.03 ± 0.21	1.03 (0.68–1.56)	0.895
Louse infestation			
(Intercept)	−0.57 ± 0.11		
Absent	Reference		
Present	0.16 ± 0.92	1.17 (0.15–7.16)	0.862
Geoclimatic zone			
(Intercept)	−0.98 ± 0.13		
Up-mid-country wet zone	Reference		
Low-country wet zone	1.67 ± 0.28	5.31 (3.12–9.25)	**<0.001**
Low-country dry zone	1.32 ± 0.34	3.75 (1.93–7.43)	**<0.001**
*B. vogeli*			
Age (months)			
(Intercept)	−2.42 ± 0.33		
Age (months)	−0.01 ± 0.01	0.99 (0.97–1)	**0.049**
Sex			
(Intercept)	−3.42 ± 0.41		
Female	Reference		
Male	0.67 ± 0.5	1.95 (0.76–5.66)	0.184
Breed group			
(Intercept)	−3.04 ± 0.3		
Local	Reference		
Exotic	0.31 ± 0.45	1.37 (0.55–3.32)	0.488
Neutering status			
(Intercept)	−3.68 ± 0.72		
Neutered	Reference		
Entire	0.82 ± 0.76	2.28 (0.63–14.55)	0.278
Tick infestation			
(Intercept)	−3.27 ± 0.32		
Absent	Reference		
Present	0.75 ± 0.47	2.11 (0.82–5.38)	0.114
Flea infestation			
(Intercept)	−2.91 ± 0.34		
Absent	Reference		
Present	−0.12 ± 0.47	0.88 (0.35–2.27)	0.791
Louse infestation			
(Intercept)	−3.09 ± 0.25		
Absent	Reference		
Present	2.69 ± 0.95	14.71 (1.85–94.55)	**0.004**
Geoclimatic zone			
(Intercept)	−3.5 ± 0.34		
Up-mid-country wet zone	Reference		
Low-country wet zone	1.37 ± 0.5	3.95 (1.44–10.71)	**0.006**
Low-country dry zone	1.28 ± 0.63	3.58 (0.93–11.59)	**0.042**
*H. canis*			
Age (months)			
(Intercept)	−1.52 ± 0.19		
Age (months)	0 ± 0	1 (1–1.01)	**0.088**
Sex			
(Intercept)	−1.41 ± 0.18		
Female	Reference		
Male	0.21 ± 0.24	1.24 (0.77–2.01)	0.383
Breed group			
(Intercept)	−1.22 ± 0.15		
Local	Reference		
Exotic	−0.27 ± 0.26	0.77 (0.46–1.26)	0.3
Neutering status			
(Intercept)	−0.99 ± 0.25		
Neutered	Reference		
Entire	−0.41 ± 0.29	0.67 (0.38–1.18)	0.156
Tick infestation			
(Intercept)	−1.52 ± 0.16		
Absent	Reference		
Present	0.62 ± 0.26	1.86 (1.12–3.06)	**0.015**
Flea infestation			
(Intercept)	−1.35 ± 0.19		
Absent	Reference		
Present	0.08 ± 0.25	1.08 (0.67–1.77)	0.746
Louse infestation			
(Intercept)	−1.28 ± 0.12		
Absent	Reference		
Present	−0.1 ± 1.12	0.9 (0.05–6.2)	0.928
Geoclimatic zone			
(Intercept)	−1.54 ± 0.15		
Up-mid-country wet zone	Reference		
Low-country wet zone	0.73 ± 0.29	2.07 (1.16–3.65)	**0.013**
Low-country dry zone	0.66 ± 0.37	1.94 (0.9–3.96)	0.077
Overall VBP infection			
Age (months)			
(Intercept)	0.2 ± 0.15		
Age (months)	0 ± 0	1 (1–1.01)	**0.042**
Sex			
(Intercept)	0.22 ± 0.15		
Female	Reference		
Male	0.43 ± 0.2	1.54 (1.03–2.3)	**0.036**
Breed group			
(Intercept)	0.32 ± 0.12		
Local	Reference		
Exotic	0.34 ± 0.21	1.41 (0.93–2.15)	0.11
Neutering status			
(Intercept)	0.48 ± 0.23		
Neutered	Reference		
Entire	−0.06 ± 0.26	0.94 (0.56–1.55)	0.809
Tick infestation			
(Intercept)	0.27 ± 0.12		
Absent	Reference		
Present	0.4 ± 0.23	1.49 (0.95–2.33)	0.082
Flea infestation			
(Intercept)	0.33 ± 0.15		
Absent	Reference		
Present	0.1 ± 0.21	1.11 (0.74–1.66)	0.623
Louse infestation			
(Intercept)	0.38 ± 0.1		
Absent	Reference	–	
Present	15.18 ± 650.87		0.981
Geoclimatic zone			
(Intercept)	−0.01 ± 0.11		
Up-mid-country wet zone	Reference		
Low-country wet zone	2.45 ± 0.44	11.58 (5.27–30.57)	**<0.001**
Low-country dry zone	1.28 ± 0.39	3.58 (1.72–8.2)	**0.001**

Bold values signify the statistically significant predictors.

**Table 4:** Route and frequency of administration (A), preparations of ectoparasiticides (B), and the overall compliance of ectoparasiticide usage by the pet owners (C) within this study.

A. Frequency of administration														
	Less than 1 month		1–3 months	4–6 months	More than 6 months	When needed		When bathing		Other		Unknown		Total

Topical	2		8	7	0	48		8		2		44		119
Systemic	1		2	2	4	15		0		2		29		55
Topical + systemic	0		0	2	0	0		0		0		1		3
Unknown	0		0	0	0	0		0		2		10		12
Total	3		10	11	4	63		8		6		84		189

B. Ectoparasite preparations														

Acaricide		Preparations used by the dog owners					Topical		Systemic		*n*		%	

Fipronil		spot-on, spray					✓				8		4.1	
Ivermectin		subcutaneous injection							✓		20		10.26	
Afoxolaner		oral tablet							✓		37		18.97	
Amitraz		solution					✓				10		5.13	
Propoxur		powder					✓				60		30.77	
Herbal products		powder, shampoo, solution					✓				6		3.08	
Unknown preparation		oral tablet, powder, shampoo, solution, spot-on, spray									54		27.69	

C. Ectoparasiticide treatment compliancy														

Compliancy status							*n*				%			

Complete							1				0.2			
Moderate							19				4.5			
Minimal							169				40			
None							234				55.3			

**Table 5:** Summary of univariable statistical associations of vector-borne pathogen (VBP) and clinical manifestations in study dogs.

Clinical sign (*n* = number of dogs with the clinical sign)/VBP	Odds ratio (95% CI)	*P*-value^†^
Any clinical sign of VBP infection^‡^ (*n* = 144)		
*A. platys*	1.5 (0.6–4.2)	0.403
*E. canis*	2.8 (1.1–7.1)	**0.025**
Haemotropic mycoplasmas	0.6 (0.3–1.3)	0.217
*B. gibsoni*	1.4 (0.9–2.1)	0.126
* B. vogeli*	1 (0.4–2.5)	0.944
*H. canis*	1.5 (0.9–2.4)	0.091
Overall VBP	1.5 (1–2.3)	**0.046**
Mono infection	1.4 (0.9–2.2)	0.054
Mixed infection	2 (1.1–3.4)	0.054
Pale mucosae (*n* = 97)		
*A. platys*	1.8 (0.6–5.3)	0.346
*E. canis*	2.3 (0.9–6)	0.097
Haemotropic mycoplasmas	0.7 (0.3–1.6)	0.423
*B. gibsoni*	1.6 (1–2.6)	**0.036**
* B. vogeli*	0.4 (0.1–1.5)	0.180
*H. canis*	1.3 (0.7–2.3)	0.306
Overall VBP	1.6 (1–2.6)	0.063
Mono infection	1.4 (0.9–2.4)	0.111
Mixed infection	1.9 (1–3.6)	0.111
Pyrexia (*n* = 39)		
Haemotropic mycoplasmas	0.8 (0.2–3.6)	1.000
*B. gibsoni*	0.9 (0.4–2.1)	0.765
*B. vogeli*	2.9 (0.5–16.1)	0.253
*H. canis*	2.4 (0.9–7)	0.093
Overall VBP	1.6 (0.6–4.7)	0.373
Mono infection	1.3 (0.4–4)	0.292
Mixed infection	2.7 (0.7–10.2)	0.292
Peripheral lymphadenopathy (*n* = 26)		
*A. platys*	2.4 (0.5–11.2)	0.249
*E. canis*	4.1 (1.2–13.4)	**0.034**
Haemotropic mycoplasmas	0.7 (0.2–3.1)	1.000
*B. gibsoni*	2 (0.9–4.5)	0.083
*B. vogeli*	0.8 (0.1–6.6)	1.000
*H. canis*	1.3 (0.5–3.3)	0.526
Overall VBP	2.5 (1–6.4)	**0.048**
Mono infection	2.2 (0.8–5.9)	0.085
Mixed infection	3.3 (1.1–9.9)	0.085
Low BCS (*n* = 72)		
*A. platys*	1.3 (0.3–4.8)	0.720
*E. canis*	3.3 (1.3–8.6)	**0.015**
Haemotropic mycoplasmas	1 (0.5–2.3)	0.943
*B. gibsoni*	1.1 (0.7–1.9)	0.617
*B. vogeli*	1.1 (0.3–3.3)	1.000
*H. canis*	1.3 (0.7–2.3)	0.457
Overall VBP	1.6 (0.9–2.8)	0.093
Mono infection	1.5 (0.9–2.8)	0.226
Mixed infection	1.7 (0.9–3.5)	0.226
Reduced appetite or anorexia (*n* = 38)		
Haemotropic mycoplasmas	0.6 (0–10.4)	1.000
*B. gibsoni*	3.1 (1–10)	0.055
*H. canis*	2.9 (0.6–15.2)	0.291
Overall VBP	2.7 (0.9–8)	0.070
Splenomegaly or hepatomegaly (*n* = 11)		
*A. platys*	4.6 (0.9–23.4)	0.104
*E. canis*	4.2 (0.8–21.5)	0.116
*B. gibsoni*	1.2 (0.4–4.1)	0.763
*B. vogeli*	3.7 (0.7–18.5)	0.142
*H. canis*	1.2 (0.3 - 4.6)	0.729
Overall VBP	2.3 (0.5–10.9)	0.348
Mono infection	1.8 (0.4–9.7)	0.370
Mixed infection	3.3 (0.6–18.8)	0.370
Presence of wounds (*n* = 48)		
*E. canis*	0.4 (0–3.2)	0.723
Haemotropic mycoplasmas	2.7 (1.2–5.9)	**0.037**
*B. gibsoni*	0.7 (0.4–1.4)	0.353
*B. vogeli*	0.8 (0.2–3.6)	1.000
*H. canis*	1.7 (0.9–3.3)	**0.046**
Overall VBP	1 (0.6–1.9)	0.956
Mono infection	1(0.5–1.9)	0.933
Mixed infection	1.1 (0.5–2.6)	0.933

^†^Significance considered at *p* ≤ 0.05; ^‡^Signs of uncomplicated VBP infection—pale mucous membranes, reduced appetite/anorexia, fever, lymphadenomegaly, splenomegaly, and hepatomegaly. Bold values signify the statistically significant associations.

**Table 6:** Odds ratios and coefficient estimates of the generalised linear mixed models and ordered logistic regression model for vector-borne pathogen (VBP) infection in Sri Lankan pet dogs.

Pathogen/Predictors	Fixed effects			Random effects				
	Estimates ± SE^†^	Odds Ratios (95% CI^‡^)	*P*-value^§^	*σ* ^2^	*τ*00 dog clinics^*α*^	ICC^¶^	*n*	Marginal R^2/^Conditional R^2^
*B. gibsoni*								
(Intercept)	0.27 ± 0.89	1.22 (0.29–5.07)	0.787	3.29	3.31	0.5	402	0.012/0.508
Age (years)	2.55 ± 0.03	1.08 (1.02–1.14)	0.011					
Haemotropic mycoplasmas								
(Intercept)	–7.51 ± 0.01	0 (0.00–0.01)	<0.001	3.29	0		384	0.287/0.304
Breed								
Exotic breeds		Reference						
Local breeds	3 ± 2.27	4.52 (1.69–12.1)	0.003					
Sex						0.02		
Female sex		Reference						
Male sex	3.53 ± 1.92	4.5 (1.95–10.37)	<0.001					
Flea infestation								
Fleas absent		Reference						
Fleas present	2.08 ± 0.99	2.37 (1.05–5.36)	0.038					
*H. canis*								
(Intercept)	–7.94 ± 0.03	0.13 (0.08–0.22)	<0.001	3.29	0		381	0.071/NA
Age (years)	2.17 ± 0.04	1.07 (1.01–1.14)	0.03					
Tick infestation								
Ticks absent		Reference						
Ticks present	1.79 ± 0.7	1.64 (0.95–2.82)	0.074					
Geo-climatic zone								
Up-mid country wet		Reference						
Low country wet	2.61 ± 0.7	2.26 (1.22–4.15)	0.009					
Low country dry	1.87 ± 1.08	2.35 (0.96–5.77)	0.062					
*A. platys*								
(Intercept)	–4.21 ± 0.05	0.01 (0–0.03)	<0.001	3.29	0		393	0.167/NA
Tick infestation								
Ticks absent		Reference						
Ticks present	1.46 ± 0.64	4.3 (1.23–15.00)	0.022					
Louse infestation								
Lice absent		Reference						
Lice present	3.15 ± 0.993	23.36 (3.34–163.44)	0.002					
Overall VBP infection								
(Intercept)	1.79 ± 8.28	7.41 (0.83–66.17)	0.073	3.29	6.23	0.65	402	0.015/0.660
Age (years)	3.32 ± 0.03	1.1 (1.04–1.17)	0.001					
Uninfected, single, or mixed infection by VBPs								
(Intercepts)							381	
No infection | Single infection	0.62 ± 0.21							
Single infection | Mixed infection	2.86 ± 0.26							
Age (years)	2.78 ± 0.03	1.08 (1.02–1.13)	0.006					
Flea infestation								
Fleas absent		Reference						
Fleas present	2.66 ± 0.36	1.74 (1.16–2.63)	0.008					
Louse infestation								
Lice absent		Reference						
Lice present	1.8 ± 9.05	7.89 (1.10–155.61)	0.073					
Geo-climatic zone								
Up-mid country wet		Reference						
Low country wet	6.35 ± 1.76	6.35 (3.72–11.01)	<0.001					
Low country dry	4.86 ± 2.67	6.78 (3.16–14.87)	<0.001					

^†^Standard error. ^‡^Confidence interval with 95% confidence. ^§^Significance considered at 0.05. ^¶^Interclass correlation coefficient. ^*α*^Variance of the intercepts of the random effects of dog clinics.

## Data Availability

Data used for this study can be found within this article and in the supplementary material.

## Supplementary Materials

Supplementary 1 Supplementary material on sample size determination, sample descriptives, and statistical analyses. Table S1: determination of the sample size using “epiR” package in R and “Epitools” web platform. Table S2: variables used to infer predictors of tick-borne pathogen infections in Sri Lankan pet dogs. Table S3: age-group, sex, neuter-status, breed group, ectoparasiticides usage, tick, and flea infestation and geoclimatic zone of the canine population included in this study. Table S4: ectoparasite prevention compliance scoring method used by considering the product, frequency of administration and duration from the last treatment.

Supplementary 2 Structured questionnaire used to obtain animal data in this study.
