# Supplementary material for: Prevalence, Distribution, and Factors Associated with Vector-Borne Pathogen Infections in Pet Dogs from Different Geoclimatic Zones in Sri Lanka
Source: Transbound Emerg Dis. 2023 Nov 13;2023:9467314. doi: 10.1155/2023/9467314 (PMC12016779; doi:10.1155/2023/9467314)
Supplement: Supplementary 1 — Supplementary material on sample size determination, sample descriptives, and statistical analyses. Table S1: determination of the sample size using “epiR” package in R and “Epitools” web platform. Table S2: variables used to infer predictors of tick-borne pathogen infections in Sri Lankan pet dogs. Table S3: age-group, sex, neuter-status, breed group, ectoparasiticides usage, tick, and flea infestation and geoclimatic zone of the canine population included in this study. Table S4: ectoparasite prevention compliance scoring method used by considering the product, frequency of administration and duration from the last treatment. [file 9467314.f1.docx]

# Supplementary A

## Table A1 Determination of the sample size using ‘epiR’ package in R and ‘Epitools’ web platform.

| Pathogen | Level of confidence % | Assumed true prevalence | Assumed diagnostic test sensitivity | Assumed diagnostic test specificity | Population size (assumed) | Precision | **Sample size to estimate prevalence** | **Required sample size to demonstrate freedom of disease (75% sensitivity)** | Confidence level with 75% sensitivity that the disease has been detected |
| --- | --- | --- | --- | --- | --- | --- | --- | --- | --- |
| *Anaplasma platys* | 95 | 0.2 | 0.75 | 0.95 | 2000000 | 0.05 | **246** | **19** | 0.954 |
| *Babesia gibsoni* | 95 | 0.15 | 0.75 | 0.95 | 2000000 | 0.05 | **196** | **26** | 0.955 |
| *Babesia vogeli* | 95 | 0.01 | 0.75 | 0.95 | 2000000 | 0.01 | **380** | **399** | 0.95 |
| *Ehrlichia canis* | 95 | 0.2 | 0.75 | 0.95 | 2000000 | 0.05 | **246** | **19** | 0.954 |
| *Hepatozoon canis* | 95 | 0.02 | 0.75 | 0.95 | 2000000 | 0.02 | **188** | **199** | 0.951 |
| *Mycoplasma haemocanis* | 95 | 0.12 | 0.75 | 0.95 | 2000000 | 0.05 | **162** | **32** | 0.951 |

**Table A2.** Variables used to infer predictors of tick-borne pathogen infections in Sri Lankan pet dogs.

| **Variable** |  |
| --- | --- |
| Breed group | - Local breed and their crosses - Exotic breeds and their crosses |
| Age | - In years |
| Sex | - Male - Female |
| Neuter status | - Neutered - Entire |
| Tick infestation | - Ticks present - Ticks absent |
| Flea infestation | - Fleas present - Fleas absent |
| Louse infestation | - Lice present - Lice absent |
| Ectoparasiticide usage and compliance | - Poor - Minimal - moderate - Good-Excellent |
| Geo-climatic zone | - Up-mid-country wet - Low-country wet - Low-country dry |

**Table A3.** Age-group, sex, neuter-status, breed group, ectoparasiticides usage, tick, and flea infestation and geoclimatic zone of the canine population included in this study.

|  |  | **Geoclimatic zone** | | | **Total** |
| --- | --- | --- | --- | --- | --- |
|  |  | **Up-mid country wet zone** | **Low country wet zone** | **Low country dry zone** |  |
| **Variables** | | | | | |
| Age group | <6 months | 24 | 1 | 3 | 28 |
|  | 6-12 months | 49 | 22 | 6 | 77 |
|  | >1-2 years | 46 | 12 | 11 | 69 |
|  | >2-6 years | 80 | 21 | 15 | 116 |
|  | >6-11 years | 74 | 14 | 3 | 91 |
|  | ≥ 12 years | 19 | 2 | 0 | 21 |
|  | Unknown | 15 | 3 | 3 | 21 |
| Sex | Female | 143 | 27 | 19 | 189 |
|  | Male | 152 | 45 | 19 | 216 |
|  | Unknown | 12 | 3 | 3 | 18 |
| Neuter status | Intact | 223 | 62 | 28 | 313 |
|  | Neutered | 69 | 5 | 7 | 81 |
|  | Unknown | 15 | 8 | 6 | 29 |
| Breed | Exotic | 98 | 42 | 7 | 147 |
|  | Local | 204 | 30 | 31 | 265 |
|  | Unknown | 5 | 3 | 3 | 11 |
| Tick infestation | Absent | 219 | 49 | 5 | 273 |
|  | Present | 80 | 19 | 22 | 121 |
|  | Unknown | 8 | 7 | 14 | 29 |
| Flea infestation | Absent | 118 | 49 | 8 | 175 |
|  | Present | 181 | 19 | 19 | 219 |
|  | Unknown | 8 | 7 | 14 | 29 |
| Louse infestation | Absent | 301 | 63 | 27 | 391 |
|  | Present | 0 | 5 | 0 | 5 |
|  | Unknown | 6 | 7 | 14 | 27 |
| Ectoparasiticides | Given | 151 | 23 | 15 | 189 |
|  | Not given | 124 | 13 | 8 | 145 |
|  | Unknown | 32 | 39 | 18 | 89 |

**Table A4.** Ectoparasite prevention compliance scoring method used by considering the product, frequency of administration and duration from the last treatment.

| **Ectoparasiticide given?** | | **Preparation given** | **Frequency that the owner claims to administer the product** | | | **Date of the last treatment with the recommended frequency of the product** | | **Total score**  **(Compliance score)** | **Compliancy group** |
| --- | --- | --- | --- | --- | --- | --- | --- | --- | --- |
| Status | Score |  | Whether owner frequently administer the product? | Whether the frequency of administration correct according to the product used? | Score |  | Score |  |  |
| Yes | 1 | Known | Yes | Yes | 1 | within the frequency | 1 | 3 | Complete |
| Yes | 1 | Known | Yes | No | 0 | unknown/incorrect | 0 | 1 | Minimal |
| Yes | 1 | Known | No | NA | 0 | unknown/incorrect | 0 | 1 | Minimal |
| Yes | 1 | Known | No | NA | 0 | unknown/incorrect | 0 | 1 | Minimal |
| Yes | 1 | Known | No | NA | 0 | within the frequency | 1 | 2 | Moderate |
| Yes | 1 | Unknown | No | NA | 0 | unknown/incorrect | 0 | 1 | Minimal |
| No | 0 | NA | NA | NA | 0 | NA | 0 | 0 | None |
