# Supplementary material for: Prevalence, Distribution, and Factors Associated with Vector-Borne Pathogen Infections in Pet Dogs from Different Geoclimatic Zones in Sri Lanka
Source: Transbound Emerg Dis. 2023 Nov 13;2023:9467314. doi: 10.1155/2023/9467314 (PMC12016779; doi:10.1155/2023/9467314)
Supplement: Supplementary 2 — Structured questionnaire used to obtain animal data in this study. [file 9467314.f2.pdf]

Supplementary B: Structured questionnaire used to obtain animal data in this study

Date (DD/MM/YYYY)
Animal Code:
Clinic/ Hospital name
Province

- ○ Western
- ○ Central
- ○ Southern
- ○ Northern
- ○ North-western
- ○ North-central
- ○ East
- ○ Uva
- ○ Sabaragamuwa

Inclusion criteria

Has the client provided the oral consent	○ Yes	○ No
The animal has a history of regular outdoor access	○ Yes	○ No
Only one dog per owner is included in this study	○ Yes	○ No

NB - Animal can ONLY be enrolled in the study if answers were "YES" for all the inclusion criteria

Signalment of the patient

Breed
Age years months (<1 year)
Sex ○ Male ○ Female
Neutered? ○ Yes ○ No ○ Unknown

Clinical History

In the previous 12 months, has the patient shown any of the following signs?
Obtain information to the best of the client's knowledge

Exercise intolerance ○ Yes ○ No ○ Don't know

If yes,
- ☐ with exercise
- ☐ with reduced physical activity
- ☐ at rest

Cough ○ Yes ○ No ○ Don't know

Dyspnoea ○ Yes ○ No ○ Don't know

If yes,
- ☐ with exercise
- ☐ with reduced physical activity
- ☐ at rest

Skin lesions ◯ Yes ◯ No ◯ Don’t know

Weight loss ◯ Yes ◯ No ◯ Don’t know

Has the patient ever been treated for tick fever? ◯ Yes ◯ No ◯ Don’t know

Has the patient ever had epistaxis/nasal bleeding? ◯ Yes ◯ No ◯ Don’t know

Has the patient ever had seizures? ◯ Yes ◯ No ◯ Don’t know

Tick/flea and deworming treatment history of the patient

Deworming treatment

Has the dog been dewormed? ◯ Yes ◯ No ◯ Don’t know

When was the last deworming treatment given?

______ Months ______ Weeks ______ Days

Type of deworming given

- ☐ Oral
- ☐ Injectable
- ☐ Topical ‘spot-on’
- ☐ Don’t know

Tick/flea treatment

Has the dog been treated for ticks/fleas? ◯ Yes ◯ No ◯ Don’t know

When was the last tick/flea treatment given?

______ Months ______ Weeks ______ Days

Type of tick/flea treatment given

- ☐ Oral
- ☐ Injectable
- ☐ Topical spray
- ☐ Topical spot-on
- ☐ Topical powder
- ☐ Topical shampoo/soap
- ☐ Collar
- ☐ Other
- ☐ Don’t know

Deworming product given (if known)

Frequency of deworming

- ☐ ≤ 2 weeks
- ☐ > 1-3 months
- ☐ Other (please specify)
- ☐ Once a month
- ☐ Once in 4-6 month
- ☐ Don't know

Comments

Anti-tick/flea product used (if known)

Frequency of tick/flea treatment

- ☐ <once a month
- ☐ >1-3 months
- ☐ Other (please specify)
- ☐ Once a month
- ☐ Once in 4-6 month
- ☐ Don't know

Comments

Physical examination

Overall condition:

○ Excellent ○ Good ○ Fair ○ Poor

Body condition score:

○ 1 (emaciated) ○ 2 (thin) ○ 3 (ideal) ○ 4 (overweight) ○ 5 (obese)

Demeanour:

☐ Bright ☐ Alert ☐ Depressed ☐ Moribund

Other (e.g. seizures, unconscious), please specify):

Rectal temperature (°C)

Skin/ Hair coat *(indicate lesions in the diagram)*

Nodules (focal/multifocal) ☐ ☐	☐ Present	☐ Absent
Growths(focal/multifocal) ☐ ☐	☐ Present	☐ Absent
Erythema	☐ Present	☐ Absent
Pruritis	☐ Present	☐ Absent
Hyperpigmentation	☐ Present	☐ Absent

Skin thickness ☐ Increased ☐ Normal ☐ Decreased

Alopecia ☐ Absent ☐ Focal ☐ Multifocal ☐ Generalised

Subcutaneous oedema ☐ Absent ☐ Present *Oedema location/s:*

Other significant skin pathology

Distribution of skin lesions

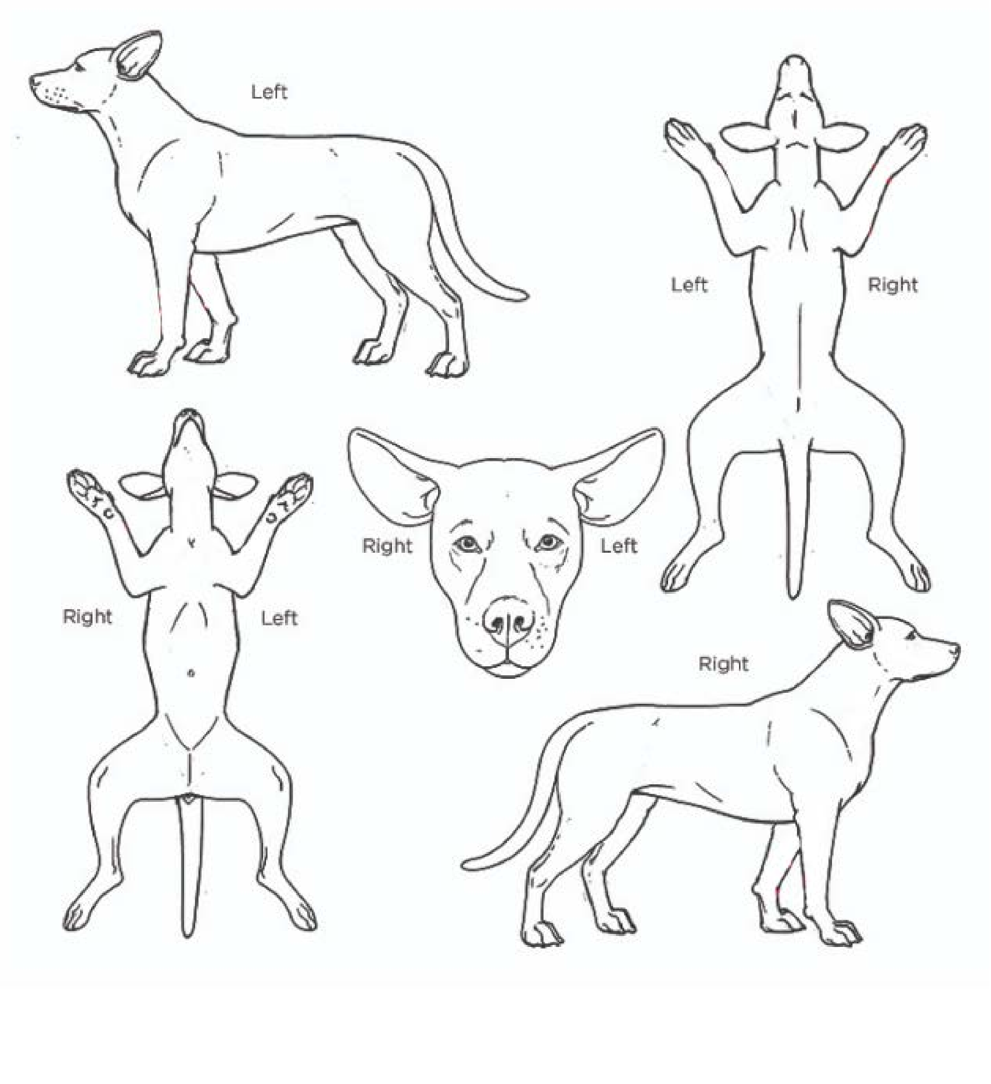

Ticks ◯ Present ◯ Absent ◯ Unsure

Fleas ◯ Present ◯ Absent ◯ Unsure

Lice ◯ Present ◯ Absent ◯ Unsure

Other ectoparasites (please specify) ◯ Present ◯ Absent ◯ Unsure

Other ectoparasites:

Eyes ◯ No abnormalities ◯ Abnormalities present

Please describe the abnormalities (e.g. hyphaema, uveitis, corneal opacity), if present:

Ears ◯ No abnormalities ◯ Abnormalities present

Please describe the abnormalities (e.g. aural haematoma, otitis externa), if present:

Nares ◯ No abnormalities ◯ Abnormalities present

Please describe the abnormalities (eg: epistaxis), if present:

Mucous membrane

◯ Normal

◯ Icteric

◯ Others (please specify)

◯ Pale

◯ Petechiae present

Superficial lymph nodes

Lymphadenomegaly ◯ Absent ◯ Localised ◯ Generalised

If present and localised,

Symmetry ◯ Unilateral ◯ Bilateral

Lymph nodes involved Mandibular ☐ Axillary ☐ Popliteal ☐ Inguinal ☐ Superficial cervical ☐

Others (Please specify)

Thoracic examination

Respiratory rate ◯ Normal ◯ Increased ◯ Decreased

Respiratory effort ◯ Normal ◯ Forced

Respiration depth ◯ Normal ◯ Shallow ◯ Deep

Cough ◯ Present ◯ Absent Sneezing ◯ Present ◯ Absent

Snoring ◯ Present ◯ Absent

Other (please specify)

Abdomen/ pelvis/ perineal examination

Ascites ◯ Present ◯ Absent

Palpable organomegaly ◯ Absent ◯ Hepatomegaly ◯ Splenomegaly

Other:

Neurological manifestations ○ Present ○ Absent

Please specify the manifestations (ex: ataxia, tremors, seizures, nystagmus) in brief if present,

Comments

**Additional history/diagnoses:**
Please report any adverse events (if no adverse events occur write N/A) **COMPULSORY**
Please tick all that apply based on sample collection, recording and storage **(COMPULSORY)**<br>☐ Whole blood sample collected in ETDA tube<br>☐ Ectoparasites in Eppendorf tubes<br>☐ Dog code noted on each sample<br>☐ All samples stored in the freezer for pick up

Veterinarian: Signature:

Notes
